# Body Builder's Shoulder: Posterior Labrum Periosteal Sleeve Avulsion (POLPSA) and Glenoid Posterior Rim Stress Fracture due to Intense Bench Pressing

**DOI:** 10.1155/2022/4533576

**Published:** 2022-01-25

**Authors:** Giovanni Bonaspetti, Giovanni Dib, Flavio Azzola

**Affiliations:** Department of Orthopaedics and Trauma Surgery, Clinical Institute S. Anna GSD, Via del Franzone 31 25127 Brescia, Italy

## Abstract

**Background:**

Shoulder overuse, both occupational and sports-related, is a major cause of shoulder pain with an estimated incidence of 0.9%-2.5% in the general population and a prevalence of 7%-27% in Europe and United States. We report on a young amateur bodybuilder presenting with a complex shoulder overuse lesion. A posterior labrum periosteal sleeve avulsion (POLPSA) with a chondral lesion of the posterior glenoid cavity and a SLAP lesion was diagnosed. *Case presentation*. A 33-year-old male construction worker complained of 9 months worsening right shoulder pain. He was an amateur body builder who would bench press heavy weights (up to 170 kg). A magnetic resonance arthrogram showed a posterior labrum sleeve avulsion, a stress chondral fracture of the posterior glenoid cavity and a SLAP lesion. Arthroscopic repair of the bicipital anchor, posterior labrum fixation and removal of the chondral fragment, proved successful and allowed the patient to return to his previous sports activity.

**Conclusions:**

Bench press creates major forces along the anteroposter axis of the upper limbs, pushing the humeral head posteriorly and increasing joint reaction force on the posterior glenoid quadrant considerably as the scapula is locked resting on the bench. This may result in a tendency for the humeral head to subluxate posteriorly which, aggravated by the high number of repetitions, puts the posterior labrum and capsula under very high stress eventually leading to labrum failure. Arthroscopic repair was shown to restore shoulder function in these athletes.

## 1. Introduction

Shoulder overuse, both occupational and sports-related, is a major cause of shoulder pain with an estimated incidence of 0.9%-2.5% in the general population and a prevalence of 7%-27% in Europe and United States [1–3]. Prolongued exposure to repeated strains and use of heavy weights may put the shoulder joint under considerable stress eventually leading to shoulder dysfunction. This is particularly true for overhead athletes such as baseball pitchers, tennis players, or handball players, with a prevalence as high as 43.5% in these groups [4, 5]. Overuse pathologies involving the wrist and the acromioclavicular joint have been described in body building but this sport is less frequently associated with shoulder joint pathology [6–8]. We report on a young amateur bodybuilder presenting with a complex shoulder overuse lesion. A posterior labrum sleeve avulsion, a stress chondral fracture of the posterior glenoid cavity and a SLAP lesion, was diagnosed.

## 2. Case Presentation

**Figure 1:**
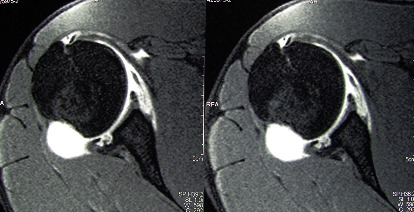
FS-PD-TSE MRI sequences depicting posterior labrum avulsion and posterior chondral lesion with a fatigue fracture of the subchondral bone and degenerative cyst.

A 33-year-old male construction worker presented to our clinic complaining of 9 months worsening right shoulder pain. He was an amateur body builder who bench pressed heavy weights (up to 170 kg). However, in the last 9 months, bench pressing became increasingly painful, forcing him to decrease his training load. No previous shoulder trauma was reported. On examination, passive and active shoulder ROM was normal, and shoulder instability tests were negative, while lift-off and biceps load tests were painful. The acromioclavicular joint was not painful on stress or palpation. The main symptom reported by the patient was a worsening shoulder pain when bench pressing with high loads. He underwent a magnetic resonance arthrogram of his shoulder showing a type 2 SLAP lesion, a large lesion of the posterior glenoid labrum and a glenoid chondropathy (type 2 ICRS) with a stress chondral fracture of the posterior glena progressed into subchondral bone erosion with cyst formation (Figure 1).

**Figure 2:**
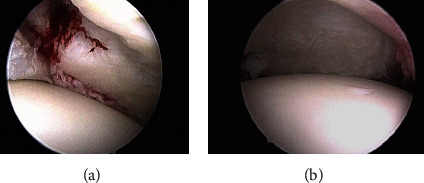
Posterior glenoid labrum avulsion (a) and glenoid chondropathy (b).

He was a candidate to arthroscopic repair: removal of the chondral fragment, repair of the bicipital anchor with a 3.5 mm suture anchor (Twin-Fix ETC Smith & Nephew), refreshing of the posterior glenoid edge, and subsequent posterior labrum fixation with a 5 mm suture anchor (Twin-Fix ETC Smith & Nephew). Notably, the same lesions described on MRI were confirmed on arthroscopy, and no further lesions, such as impact fractures of the humeral head (Hilhyl-sachs or McLaughlin type), were noted (Figure 2).

After 3 months of physical rehabilitation, the patient was able to resume his normal life. Six months postoperatively, he resumed gym training despite medical advice against it. Two years postoperatively, he was still bench pressing his maximal weight.

## 3. Discussion

Bench pressing biomechanics have been extensively reviewed in the pertinent literature; however, some further considerations are worthy of note.

When bench pressing, the athlete lies flat on his/her back on the bench and holds the weight bar with both hands aligned on the scapular plane. The exercise can be broken down into 5 different phases:

Phase 1: set up: the athlete's body lies on the bench on a 5-points contact position in which the back of the head, the shoulder blades (scapular plane), the gluteals, and the feet remain in contact with the bench or floor.

Phase 2: unrack: grasping the bar with an opposing thumb grip, the athlete lifts the barbell and positions it over his chest.

Phase 3: descent: the athlete lowers the bar to his chest along a straight trajectory, while retracting both scapulae to form a more stable base of support against the bench.

Phase 4: pause: the bar is held still on top of chest, not touching it, preparing to lift.

**Figure 3:**
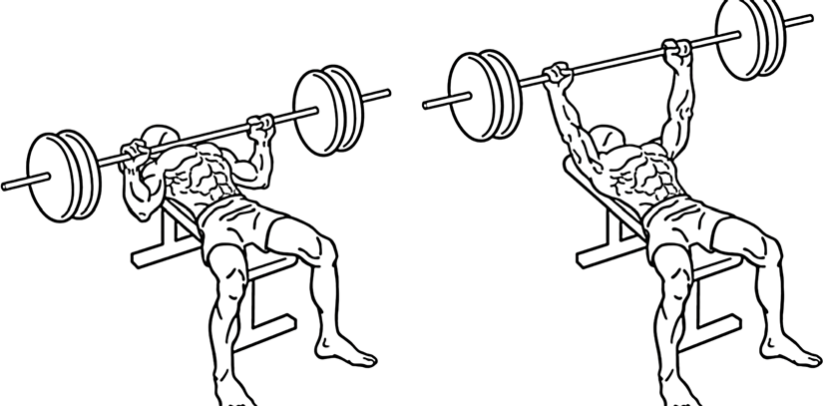
Pause and ascent phases of bench pressing.

Phase 5: ascent (and lockout): the athlete pushes the barbell upward until full arm extension, bringing it to the starting position (Figure 3).

**Figure 4:**
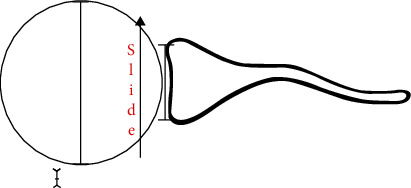
Shear forces acting on shoulder during bench pressing.

**Figure 5:**
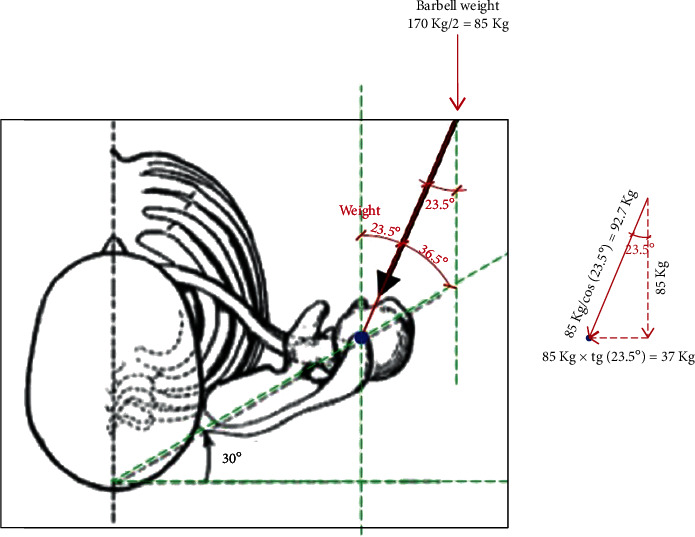
Applied force vectors on shoulder and glenoid articular surface when bench pressing 170 kg.

Bench pressing creates major forces along the anteroposter axis of the upper limbs. In the first three phases, the weight vector pushes the humeral head posteriorly, increasing the joint reaction force on the posterior glenoid quadrant considerably as the scapula is locked resting on the bench. If the scapula was free to move and not restrained, it could follow the humeral head shift by retracting further backward. A fixed scapula and the posterior push of the humeral head by the barbell weight may result in a tendency for the humeral head to subluxate posteriorly which, aggravated by the high number of repetitions, puts the posterior labrum and posterior capsule under very high stress (Figures 4 and 5).

**Figure 6:**
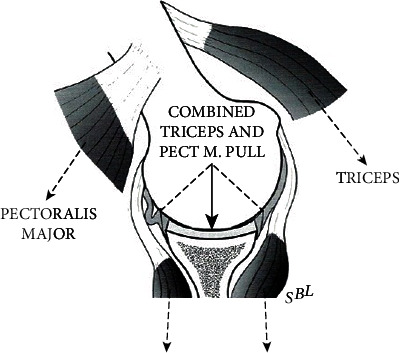
Resulting vector of forces acting on glenohumeral joint by the combined pull of triceps and pectoralis major during cocontraction in the fifth phase of bench pressing.

In the fifth phase, the pectoralis major activates to adducts the humerus against the trunk to lift the barbell, further increasing joint reaction forces on the glenoid cavity. Again, high repetitions may aggravate chondral stress and damage. Higher training weights result in greater posterior humeral head translation, joint hyper pressure, and potential damage. From an anatomical standpoint, the scapula has an angle of 30° with respect to the thoracic cage and is anteverted by 10° (Figure 5). If the hand grip on the bar is wider than shoulder width, this increases the humeral head angle of incidence with respect to the glenoid cavity, in turn increasing shear forces during posterior translation of the humeral head (Figure 5). In the current case, MRI arthrogram revealed a type 2 SLAP lesion, posterior labrum tear and chondral fracture of the posterior glenoid quadrant with a subchondral degenerative bone cyst. The patient was an amateur body builder who used to bench press up to 170 kg with no history of trauma. Initially, the pain was well managed allowing the patient to maintain his level of training. Thereafter, it gradually worsened to a point where he was unable to keep training to his usual level and sought medical advice. It appears that the mechanism of the lesion was chronic and progressive, consistent with the presence of a degenerative subchondral bone cyst. This explanation would classify such a lesion in the broad spectrum of AIOS (Acquired Instability, Overstressed Shoulder) and posterior shoulder instability. In 2002, Yu et al. described the link between POLPSA and posterior instability but none of these patients had chondral or glenoid damage [9]. In contrast, our patient presented with an avulsion of the posterior labrum together with a stress fracture of the posterior glenoid rim. It is possible that the posterior labrum and capsular damage occurred during the first three phases of bench pressing, when the pectoralis major is not activated and thus not restraining the posterior shift of the humeral head. Instead, chondral glenoid damage and bone cyst formation (secondary to synovial fluid leak) may have occurred during the fifth phase, when cocontraction of the pectoralis major to lift the barbell greatly increases intrarticular pressure and cartilage wear (Figure 6).

Surgical repair proved successful and allowed the patient to return to his previous sports activity. Even though he has managed to continue lifting such heavy weight, it is not in the authors' opinion that a return to such strenuous activities is advisable.

## 4. Conclusion

To our knowledge, this is the first report of posterior chondral glenoid stress fracture and posterior labrum avulsion in an amateur body builder without a history of shoulder dislocation. Body building and bench pressing put the shoulder under considerable stress, potentially resulting in complex shoulder injury. Arthroscopic repair was shown to restore shoulder function in these athletes.

## Data Availability

Data sharing is not applicable to this article as no datasets were generated or analyzed during the current study.
